# Maternal Dietary Intakes, Red Blood Cell Indices and Risk for Anemia in the First, Second and Third Trimesters of Pregnancy and at Predelivery

**DOI:** 10.3390/nu12030777

**Published:** 2020-03-15

**Authors:** Faith Agbozo, Abdulai Abubakari, Joyce Der, Albrecht Jahn

**Affiliations:** 1 Department of Family and Community Health, School of Public Health, University of Health and Allied Health Sciences, Ho, Ghana. Private Mail Bag 31 Ho, Ghana; 2 Heidelberg Institute of Global Health, University Hospital Heidelberg Germany. Im Neuenheimer Feld 130.3, 60120 Heidelberg, Germany; albrecht.jahn@uni-heidelberg.de; 3 Public Health Department, University for Development Studies, Tamale Ghana. P. O. Box TL 1350 Tamale, Ghana; abubakari.abdulai1@uds.edu.gh; 4 Department of Epidemiology and Biostatistics, School of Public Health, University of Health and Allied Health Sciences, Ho, Ghana. Private Mail Bag 31 Ho, Ghana; jdberkumwine@uhas.edu.gh

**Keywords:** anemia in pregnancy, hemoglobin, iron deficiency anemia, dietary diversity, red blood cell, food intakes, dietary iron, micronutrients, malnutrition, Ghana

## Abstract

As anemia remains a major public health problem in Ghana, we examined the effect of dietary intakes, and antenatal care (ANC) practices on red cell indices and anemia prevalence during the pregnancy continuum for 415 women. Dietary history was taken using the Food and Agriculture Organization minimum dietary diversity indicator for women (MDD-W). Intake of ≥5 food groups was a proxy for micronutrient adequacy. Odds for anemia and meeting the MDD-W were estimated using ordinal and binary logistic regressions respectively. Intakes of 41.4% were micronutrient inadequate. At any time point in pregnancy, 54.4% were anemic (mild = 31.1%; moderate = 23.1%; severe = 0.2%) with 10%-point variation across the first (57.3%), second (56.4%) and third (53.3%) trimesters and pre-delivery (47.7%); 27.8% were anemic throughout pregnancy while 17.1% were never anemic. Morphologically, microcytic (79.4%) and hypochromic (29.3%) anemia were most prevalent, indicating nutritional deficiencies. Planning the pregnancy was a significant determinant for meeting the MDD-W. Overall, adolescence, poor diet, suboptimum ANC and underweight were associated with moderate and severe anemia. In specific time-points, dietary counselling, malaria, iron-folic acid supplementation, sickle cell disease and preeclampsia were observed. Decline of anemia during pregnancy suggests the positive impact of ANC services and supports strengthening education on dietary diversification during ANC.

## 1. Introduction 

Globally, maternal anemia remains a public health problem and progress towards reduction is marginal as 38% of women still experience anemia during pregnancy, accounting for 20% of maternal mortality [1]. Africa and Asia account for over 85% of the absolute burden [2], but 43% of pregnant women in low- and middle-income countries are anemic [2]. Like most nutrients, iron needs increase during pregnancy to support increasing expansion in hemoglobin and red cell mass, basal iron losses, growth of fetal tissues and placenta and blood loss during delivery [3]. Anemia in pregnancy increases maternal vulnerability to suboptimum gestational weight gain, antepartum and puerperal infections and postpartum hemorrhage while the newborn is likely to have shorter gestation, small-for-gestation, low birth weight and length and poor Apgar [4,5,6].

According to the Ghana demographic and health survey report, 70.0% pregnant women were anemic in 2008 (mild: 23.4%; moderate: 40.0% and severe: 6.6%) [7] and reduced to 44.6% in 2014 (mild: 20.1%; moderate: 24.0% and severe: 0.5%) [8]. The 25.4% reduction has been attributed to strengthening implementation of nutrition-specific interventions that address the immediate determinants of maternal malnutrition and fetal growth during antenatal care, particularly iron-folic acid supplementation, nutrition education, treatment of helminths, food fortification and supplementation [8,9]. In addition, attributed are nutrition-sensitive interventions that incorporate nutrition objectives with the aim to influence the underlying determinants of malnutrition such as malaria prevention, improved water, sanitation and hygiene, family planning services, agriculture and food security, women’s empowerment, among others.

In developing countries, suboptimal dietary intakes during pregnancy remain the primary cause of nutritional anemia in pregnancy [10,11]. Habitual intakes that are imbalanced in macro- and micronutrients as result of being heavily cereal- and plant-based protein-sources with little vegetables and animal products are important contributors to nutritional anemia culminating in micronutrient deficiencies, especially folate and iron [10,11]. Lack of chicken and dark green leafy vegetables in the diet are associated with two- and five-folds odds for anemia [12]. In Ghana, food intakes during pregnancy are driven by food beliefs and practices, unfounded knowledge on the functions of foods and supplements, maternal health status, physiological changes during pregnancy and access to nutrition information [13].

Evidence suggests that intake of diversified diets is a reliable measure to assess the micro- and macronutrient adequacy of intakes of women of reproductive ages [14,15]. A simple population-level tool to assess diet quality is the Food and Agriculture Organization’s (FAO) minimum dietary diversity indicator for women (MDD-W) of reproductive age (15–49 years) [16]. The MDD-W is estimated from ten food groups based on a dichotomous indicator and is summarized across eleven micronutrients that reflects diet quality as a proxy for higher micronutrient adequacy [14]. Although consumption of diversified diets of at least four food groups daily during pregnancy is significantly associated with higher hemoglobin (Hb) concentrations [11], achieving dietary diversity among disadvantaged populations is challenging. In northern Ghana, out of a mean dietary diversity score of nine, urban dwelling pregnant women (4.42 ± 0.10) had significantly higher scores compared to their counterparts in the rural dwellers (3.84 ± 0.14) [17].

In line with the second target of the 2012 World Health Assembly Resolution to reduce anemia by 50% in women of reproductive age [18], efforts have intensified in Ghana to improve maternal counselling during antenatal care (ANC), consumption of nutrient-rich diversified local foods as well as strengthen implementation of nutrition-specific maternal and newborn health interventions. On this premise, we investigate the association of maternal dietary intakes and ANC practices on anemia prevalence in the first, second and third trimesters of pregnancy and at pre-delivery and the morphology of the red blood cells.

## 2. Materials and Methods

### 2.1. Design 

In this prospective study, an association of maternal dietary intakes, anthropometric indices and antenatal care practices on Hb levels were assessed in four-time points; in the first, second and third trimesters of pregnancy and at the point of admission for labor and delivery. At the first ANC booking in the first trimester, information was taken on maternal socio-demographic status, medical and obstetric history, dietary intakes, anthropometry and Hb. Red blood cell (RBC) indices were repeated in the three subsequent time points.

### 2.2. Study Setting 

Study participants were recruited from five public hospitals in four districts in the Volta Region, Ghana, representing healthcare delivery at the primary, secondary and tertiary levels. As ANC and delivery services are provided predominantly in secondary facilities in Ghana, one primary facility, three secondary facilities and the only teaching hospital in the region were included. All the study facilities provide both basic and comprehensive emergency obstetric care. The purposeful selection was to provide an overview of maternal health care services among urban, peri-urban and rural populations and ensure that socio-demographic characteristics of the study population are similar as in the region.

Rural inhabitants constitute 66.3% of the region’s population. Females in the reproductive ages (15–49 years) represent 47% of the total 1,098,854 female population and the total fertility rate is 3.2 children per woman [19]. The region is unique for having all the ecological zones and ethnic groups in Ghana inhabiting as indigenes but has one of the poorest health indicators. The region has the highest burden of anemia (48.7%) in Ghana, above the national average (44.6%) [8]. Regarding anthropometric status, 7.2% and 9.3% of women in the region are underweight and obese, respectively. Only about a third take iron supplements for 90 days or more (38.9%) or deworming medications (32.2%) during pregnancy compared to the national average of 59.4% and 39.4% respectively [8].

### 2.3. Sample Size and Sampling 

The sample size was determined based on a target population of over 500,000 women in their reproductive age in the study area. A 95% confidence level corresponding to 1.96 alpha, an error margin of 0.05 and a default population proportion of 50% to account for lack of information on the percentage of women who achieve adequate dietary diversity during pregnancy in Ghana were used yielding a minimum sample size of 384. Almost all pregnant women in Ghana receive ANC from skilled providers but 33.4% register after three months of gestation, 12.8% record fewer than four ANC visits throughout pregnancy while health facility delivery is 73.1% [8]. Therefore, we doubled the sample size to account for participants’ drop-out. Participants were recruited consecutively, in that every pregnant woman who visited any of the five facilities during the study period whose pregnancy was less than 13 weeks old and who intended to deliver in the study facility were enrolled. An initial 807 pregnant women were recruited of which 415 participants who had complete data on RBC indices in the first, second and third trimesters and at the point of admission for labor and delivery were reported in this paper. Seventy-four percent of the participants were proportionately recruited from three secondary facilities (*n* = 598) while about 13% were each recruited from one primary ( *n*= 108) and one tertiary (*n* = 101) facility in proportion to size.

### 2.4. Data Collection 

#### 2.4.1. Dietary Intakes 

Dietary patterns were derived a priori, using a food frequency questionnaire (FFQ) designed based on typically consumed foods in Ghana. We took information on intakes of fats and oils, snacks, confectionaries, fizzy drinks, fruit juices, alcohol, smoking, non-nutritive pica, supplements as well as food cravings, aversions and taboos. The dietary data was modified into a ten-food-group FFQ according to the FAO MDD-W: staple foods (grains, white roots, tubers and plantains); pulses (beans, peas and lentils); nuts and seeds; dairy; fleshy foods (meat, poultry and fish); eggs; dark green leafy vegetables; other vitamin A-rich fruits and vegetables; other vegetables; and other fruits [16]. The FFQ had seven frequency of consumption categories ranging from at least once daily; 3–6 times per week; 1–2 times per week; 2–3 times per month; once monthly; rarely to never. The data was validated with a non-quantitative 24-h recall of foods eaten the day and night prior to the survey. Based on the FAO MDD for women of reproductive age [16], consumption of at least five out of the ten FAO-defined food groups the day prior to the survey was used as a proxy measure to assess the micronutrient adequacy of intakes of the pregnant women [14,15].

#### 2.4.2. Maternal Anthropometry 

At registration, maternal weight, height and mid-upper arm circumference (MUAC) were measured following standard WHO guidelines. Body mass index (BMI) was classified as underweight (<18.5); normal weight (18.5–24.9); overweight (25.0–29.9); and obese (≥30) [20]. Since no optimal MUAC cut-off is currently available for use during pregnancy, population-specific cut-off values based on the median was used. Values below the 10th percentile were presumed to be associated with a high risk of undernutrition, whereas values above the 90th percentile indicated obesity. At pre-delivery, weight change was determined based on weight measured at booking and at subsequent monthly ANC visits.

#### 2.4.3. Red Blood Cell Indices 

As a routine ANC practice in Ghana, Hb is measured at registration, 36 gestational weeks and at admission for delivery. The procedure usually involves the collection of venous blood which is analyzed on a hematology analyzer. Therefore, these routine surveillance data were extracted from the hand-held maternal health record. We did a full blood count between 20 to 28 gestational weeks. One milliliter of venous blood was withdrawn and analyzed on the Sysmex Europe GmbH XS-500i hematology analyzer (Bornbarch Germany), which is factory calibrated against the hemiglobincyanide method.

Red blood cell (RBC) indices measured included Hb, hematocrit (Hct), RBC count, mean corpuscular volume (MCV), mean corpuscular Hb (MCH), mean corpuscular Hb concentration (MCHC) and red cell distribution width (RDW). Diagnosis of iron deficiency anemia was based on the WHO criteria of Hb concentration <11.0 gram/decilitre (g/dL). Its severity was classified as mild (10.0–10.9 g/dL), moderate (7.0–9.9 g/dL) and severe (<7.0 g/dL) [21]. To determine other RBC abnormalities, second trimester reference ranges suggested by Abbassi-Ghanavati et al. was used: RBC count 2.81–4.49 × 10^12^/L, Hct 30%–39%, MCV 85.8–99.4 fl, MCH 30–33 pg/cell, MCHC 32.4–35.2 g/dL and RDW: 12.3%–14.7% [22].

### 2.5. Statistical Analysis 

Data were analyzed using descriptive statistics, including frequencies, interquartile ranges, means ( $\bar{x}$) and standard deviations (SD) in Stata software (version 14.2). Background characteristics of participants were described according to micronutrient adequacy of dietary intakes. Categorical variables were compared using Pearson’s chi-squared test (χ2), whereas continuous variables were compared using t-test. Post hoc analysis was conducted for categorical variables with more than two response levels using the Bonferroni correction, whereas inter-subject variability resulting from the comparison at different time points was reduced using the McNemar’s test.

We checked for multicollinearity and singularity among exposure variables. Where the correlation was >0.7, one of the variables was omitted from the multivariate model. Factors that predicted the likelihood of a pregnant woman meeting the MDD indicator were estimated in univariate and multivariate binary logistic regression models. Unadjusted odds for anemia was estimated through ordinal univariate logistic regression. Outcomes were classified as moderate, mild and non-anemic. Severe anemia was combined with moderate anemia due to small number of cases (0.2%). Incomplete cases were deleted listwise. Afterwards, we fitted a multivariate model to control for potential confounding variables. Selection of variables for the adjusted model was based on evidence of association with anemia from literature, clinical practice and correlation with the outcome variable (χ^2^ < 0.10). The model was built on the assumption that as the value of the latent variable increased, the observed outcome monotonically increased. *p*-values < 0.05 at 95% confident level (CI) were statistically significant.

### 2.6. Ethical Considerations 

The study was approved by the Ghana Health Service Ethics Review Committee (GHS-ERC-GM 04/02/16) and the Institutional Review Board of the Heidelberg University Medical Faculty (S-042/2016). Teenagers were included as they were ethically considered as emancipated adults. All participants provided written informed consent, either by signing or thumb-printing the consent form as evidence of willingness to participate.

## 3. Results 

### 3.1. Dietary Diversity 

Diets of 58.6% (*n* = 243, 95% CI: 54.2–63.4) of the 415 participants contained at least five food groups thus meeting the MDD indicator, whereas diets of the remaining 41.4% (*n* = 172, 95% CI: 36.6–45.8) contained less than five food groups and were therefore classified as micronutrient inadequate. Out of ten food groups, mean MDD score was 5.08 food groups (standard deviation (SD = 1.82)). Mean MDD score for the micronutrient adequate and inadequate groups were 5.86 (SD = 1.63) and 3.99 (SD = 1.49) food groups, respectively (*p* < 0.0001).

### 3.2. Socio-Demographic, Health and Anthropometry Characteristic 

Presented in Table 1 is a description of the study participants according to the adequacy of micronutrient intakes. Higher proportion of women whose intakes were micronutrient poor received ANC care in primary facilities (9.1% vs. 24.4%, *p* < 0.0001), did not plan the index pregnancy (32.2% vs. 43.8%, *p* = 0.012) and had more helminths (0.0% vs. 10.3%, *p* = 0.021) but fewer had hepatitis B infection (6.3% vs. 1.1%, *p* = 0.047).

### 3.3. Habitual Dietary Patterns 

Daily meals were mostly prepared from corn accompanied by fish and vegetables. Eggs and dairy products were combined with the fleshy foods group. Corn (70.6%) and rice (41.2%) were the most daily consumed staple. Fish (84.9%) and poultry (21.6%) were the most consumed fleshy food. Egg and milk were consumed by 20.5% and 17.6% participants daily. Groundnut was the most daily consumed (21.6%) nuts. Pulses were the least consumed group with black eye beans (11.0%) being the most consumed in that group. About one-fourth of the participants took dark green leafy vegetables daily. The main varieties were ‘kontomire’ (cocoyam/taro leaves), ‘gboma’ (African eggplant leaves) and ‘ayoyo’ (Corchorus leaves). Intake of other vitamin A-rich vegetables, particularly chilli pepper (83.4%) and tomatoes (74.2%) followed a similar pattern as for the staple foods because they are usual accompaniments. Banana and orange, which were the most consumed fruits, were eaten by about a third daily. While smoking and intake of alcohol were rare, daily consumption of sweetened foods (35.7%) and beverages (21.1%) was relatively high. Overall, 17.7% adhered to some food taboos and 18.8% had some food aversions. Foods tabooed were mainly high iron bioavailable animal food sources (pork, mutton, mudfish, catfish, crab, snail, beef) and okro. Meals averted included beans, cassava flour, fermented corn products, eggs, fresh fish, oily nuts (particularly groundnuts and palm nuts) and alcohol. Although 24.0% had peculiar food cravings, apart from one pregnant woman who ate white clay, all the foods craved for were healthy.

### 3.4. Predictors for Meeting the Minimum Dietary Diversity Indicator 

In the univariate analysis, informal sector employment (UOR: 1.93, 95% CI: 1.16–3.21, *p* = 0.011), receiving ANC in a secondary facility (UOR: 7.32, 95% CI: 4.10–13.09 *p* < 0.0001) and planning the index pregnancy (UOR: 1.77 95% CI: 1.19–2.61, *p* = 0.004) predicted eating according to the FAO MDD-W. In the multivariate analysis, ANC in a secondary facility (AOR: 5.12, 95% CI: 2.12–12.37, *p* < 0.0001) and having planned the index pregnancy (AOR: 2.31, 95% CI: 1.07–4.92, *p* = 0.031) remained the significant predictors for meeting the FAO MDD-W (Table 2).

### 3.5. Anemia Levels by Hemoglobin, Red Blood Cell Morphology and Mean Red Blood Cell Indices 

**Figure 1:**
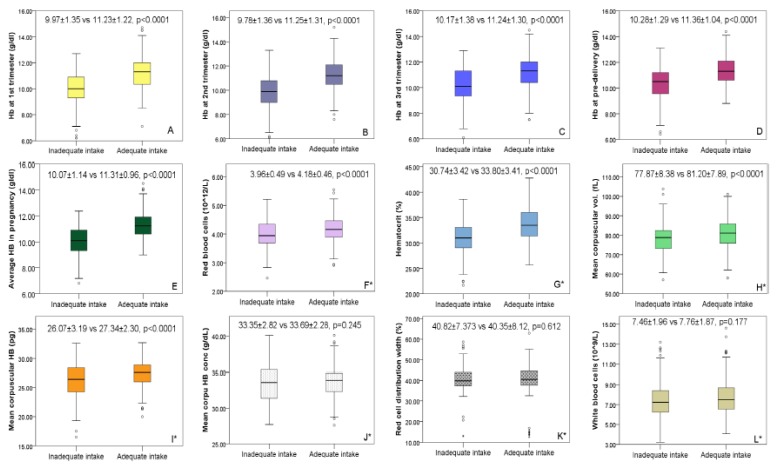
Inter-quartile and mean values for hemoglobin and other red blood cell indices classified according to the adequacy of micronutrient intake. ^*^RBC indices were measured only in the second trimester. Footnote: Hemoglobin in the (**A**) first, (**B**) second, (**C**) third trimesters of pregnancy and (**D**) at the point of admission for labor and delivery, (**E**) mean hemoglobin during pregnancy, (**F**) red blood cells (**G**) hematocrit, (**H**) mean corpuscular volume, (**I**) mean corpuscular hemoglobin, (**J**) mean corpuscular hemoglobin concentration, (**K**) red cell distribution width and (**L**) white blood cells.

**Figure 2:**
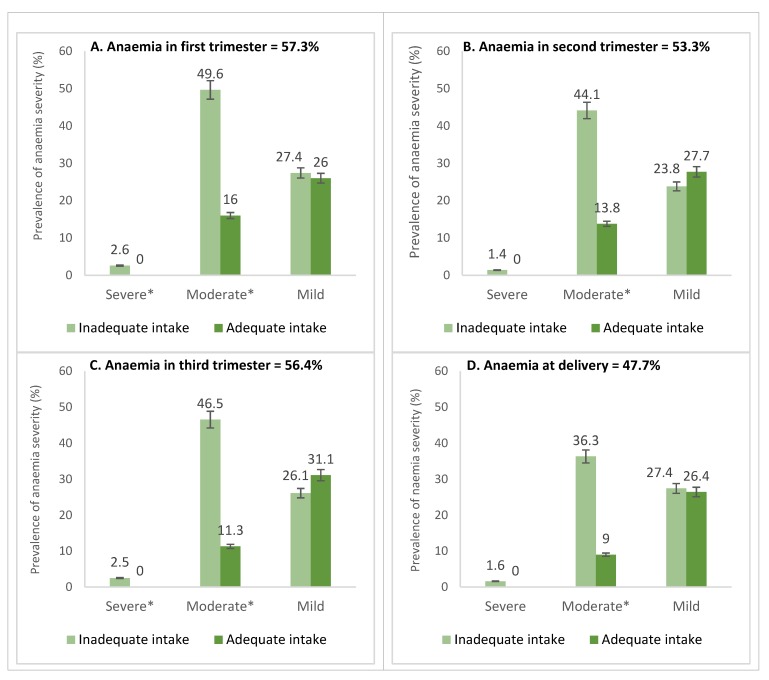
Prevalence and severity of anemia in the(**A**) first, (**B**) second and (**C**) third trimesters of pregnancy and (**D**) at the point of admission for delivery stratified according to the adequacy of micronutrient intake. Footnote: *Multiple comparison shows the categories that differed significantly (*p* < 0.05). Anemia classified as mild (10.0–10.9 g/dL), moderate (7.0–9.9 g/dL) and severe (<7.0 g/dL) (WHO, 2011).

Higher mean Hb concentrations were seen in the third trimester (10.79 ± 1.43 g/dL) and at pre-delivery (10.91 ± 1.26 g/dL) while Hb concentrations in the first (10.65 ± 1.51 g/dL) and second (10.70 ± 1.42 g/dL) trimesters were lower. Overall, the mean Hb for the four-time points was 10.80 ± 1.20 g/dl. Mean change in Hb from the first trimester to pre-delivery was 0.268 g/dL. Between the micronutrient inadequate and adequate groups, except for MCHC (33.35 ± 2.82 vs. 33.69 ± 2.28 g/dL) and RDW (40.82 ± 7.373 vs. 40.35 ± 8.12%) where no differences were found, all other RBC indices including Hct (30.74% ± 3.42% vs. 33.80% ± 3.41%), RBC (3.96 ± 0.49 vs. 4.18 ± 0.46 × 10^12^/L), MCV (77.87 ± 8.38 vs 81.20 ± 7.89 fl) and MCH (26.07 ± 3.19 vs 27.34 ± 2.30 g/dL) were significant below the 1%-level. Interquartile ranges and mean RBC indices for the micronutrient adequate and inadequate groups is presented in Figure 1. Overall, anemia prevalence was 54.4% (mild = 31.1%; moderate = 23.1%; severe = 0.2%). Except at the point of delivery where anemia was relatively lower (47.7%), anemia at first (57.3%), second (56.4%) and third (53.3%) trimesters were all above 50% (Figure 2).

At all the time points, anemia was significantly higher (*p* < 0.0001) in women whose intake was micronutrient inadequate. Only 17.1% were non-anemic at all the time points assessed, whereas 27.8% were anemic throughout pregnancy. Prevalence was significantly higher among primary (60.9%) and secondary (58.7%) facility users compared to tertiary (27.0%) facility users. Similar trends were observed in the first (primary: 64.0%; secondary: 59.7%; tertiary: 34.3%; *p* = 0.011), second (primary: 61.5%; secondary: 60.8%; tertiary: 28.8%; *p* < 0.0001) and third trimesters (primary: 48.8%; secondary: 58.1%; tertiary: 32.7%; *p* = 0.004) but not at pre-delivery (primary: 47.2%; secondary: 50.0%; tertiary: 36.6%; *p* = 0.121). Between the micronutrient inadequate and adequate groups, the proportion with mild anemia was statistically similar, but women whose intake were micronutrient inadequate experienced more moderate and severe forms of anemia. The RBC morphology showed hypochromic and microcytic and hyperchromic anemia indicating nutritional anemia mainly from iron, folic acid and vitamin B_12_ deficiencies (Table 3).

### 3.6. Risk Factors for Anemia in Pregnancy 

In the univariate analysis, higher odds for anemia were associated with maternal age below 19 years (UOR:3.69 95% CI: 1.83–7.41), being a single mother (UOR: 1.76 95% CI: 1.17–2.67), not planning the pregnancy (UOR:1.50 95% CI: 1.03–2.19), receiving ANC in lower-level facilities (UOR:3.71 95% CI: 2.05–6.73), little formal education of both the women (UOR:2.07 95% CI: 1.07–4.02) and her partner (UOR: 2.40 95% CI: 1.14–5.06), poor dietary intake (UOR: 3.30 95% 2.18–4.99), malaria infection (UOR: 3.61 95% CI: 1.58–8.25) and belonging to the AB blood group (UOR: 3.48 95% CI: 1.41–8.59) (Table S1). In addition, the fewer the ANC contacts (UOR: 0.86 95% 0.78–0.94) and the lower the BMI (UOR: 0.37 95% CI: 0.18–0.74), the higher the risk.

In the multivariate analysis (Table 4), overall, higher odds for anemia was associated with poor dietary intake (AOR: 2.73 95% CI: 1.35–5.50), whereas women who were housewives had a lower risk (AOR: 0.31 95% CI: 0.12–0.80). In addition, the lower the maternal age (AOR: 0.90 95% CI: 0.84–0.96), the fewer the ANC contacts (AOR: 0.85 95% CI: 0.75–0.96) and the lower the BMI (AOR: 0.87 95% CI: 0.82–0.94), the higher the risk. The associations were observed for moderate and severe anemia but not mild anemia. Specific risk factors for each time point are shown in Table 4.

## 4. Discussion 

Maternal anemia was a severe public health problem in the study setting as Hb of half of the participants was below 11.0 g/dL at any one time-point during pregnancy, whereas only 17% were never anemic throughout the pregnancy continuum. Globally, anemia affects over 50% of pregnant women in developing countries [23]. Adequate diet supported by iron-folic acid supplementation (IFA) is shown to reduce anemia [11], fetal growth restriction, preterm and neonatal mortality [17,24]. Planning towards the pregnancy significantly reduced the likelihood for inadequate micronutrient intakes. Studies show that women who prepare towards pregnancy become more health-conscious, and thus have better birth outcomes [25] since they are more likely to take iron-folic acid supplements before pregnancy, achieve optimal ANC attendance and adhere to health and nutrition advice given.

Morphologically, microcytic and hypochromic anemia was prevalent, being significantly higher among participants whose dietary intakes was poor, an indication of micronutrient deficiencies. A major cause is iron deficiency plus vitamin B_6_, folate and B_12_ deficiencies. However, hypochromic anemia may also be caused by hookworms which we found to be 4%. Low levels of vitamin B_12_, for instance, can lead to pernicious anemia which is common among vegetarians. No participant was a vegetarian. In fact, 85% consumed fish the day before the survey, whereas about 20% consumed poultry, eggs and milk, which are rich sources of vitamin B_12_ and folate. Sparingly consumed were iron-rich food sources, particularly legumes (<10%) and dark green leafy vegetables (≈25%). Despite reported intakes, the actual quantity consumed might be sub-optimum, reflecting in the high nutritional anemia.

Although physiological adjustment during pregnancy results in 2–3 folds increase in intestinal iron absorption [21], poor dietary diversity increases risk by 3–4 folds in all the trimesters [26]. During pregnancy, Hb concentrations change in healthy, iron-sufficient women to accommodate the increasing maternal blood volume and fetal metabolism [21], causing expansion of red cell mass and depletion of iron stores [2,27]. Typically, Hb and Hct concentrations decline in the first trimester, reaching the lowest point in the second trimester, and begin to rise again in the third trimester [21]. We noted a steady decline in anemia prevalence throughout pregnancy continuum vis-a-vis an increase reported in China (11.2%, 20.1% and 26.2% in the first, second and third trimesters respectively) [28]. A global meta-analysis in 2016 reported a 70% prevalence in Ghana [2] compared to the 54% overall prevalence we found. Effects of malaria and deworming strategies, IFA supplementation, nutrition education, referral of severely anemic women to higher facilities, blood transfusion, and provision of higher iron doses via faster intramuscular and intravenous routes could account for the downward trend.

Generally, risk for anemia was reduced significantly among women who consumed diversified diets, were older than 20 years, had lower parity, were housewives and had optimal ANC contacts. Elsewhere, lower risk for anemia were associated with higher formal education [28], large family size [29], gainful employment and high income [26,30]. Food intakes in Ghana are increasingly becoming monotonous and energy-dense and less diversified in fruits, vegetables and animal sources across the lifespan with infants [31], school-age children [32] and the elderly [33] being affected. Interestingly, 20% of the participants averted iron-rich foods; only 25% consumed dark green leaves daily, whereas pulses were the least eaten. Maternal nutrition is affected by food beliefs, socio-cultural practices and falsehood on the functions of foods and supplements [13], thereby retarding nutritional interventions. Adolescent girls are known to have a higher risk for anemia due to monthly blood loss, which could become severe during pregnancy owing to increased iron needs for the growth of both mother and developing fetus [2]. Intensifying family planning services to reduce unplanned pregnancies among adolescents and older women who risk being anemic due to high parity and closed birth spacing is crucial. Reduced risk among unemployed women, possibly signifies investments into food preparation at home compared with their employed counterparts who might buy unhealthy food while busy at work.

Regarding specific trimesters, nutrition counselling, for instance, reduced anemia in the first trimester and at delivery. Content-specific education targeted at increasing intake of iron, folic acid, vitamins A, B_12_ and C is key [11]. Counselling on hematopoietic nutrients including iron absorption enhancers (vitamin C-rich foods), inhibitors (foods high in inositol, phytates, iron-binding phenolic compounds like tannins), and practices like avoiding tea, coffee and dairy products during meals is needed. Education should also include food processing methods like soaking, fermentation, germination or thermal preparation which improves bioavailability and absorption of iron [34]. Concerning anthropometric indices, women with lower BMI had increased odds for anemia [28]. Like other studies [26,28], non-adherence to counselling on IFA increased odds for anemia at 36 weeks. Maternal undernutrition affects nutrient–nutrient interactions, immune function and metabolism and is associated with preterm delivery, small-for-gestational-age and perinatal death [4,5,35]. In addition, malaria significantly increased risk for anemia in the first and second trimesters, implying that the chemoprophylaxis for the intermittent prevention of malaria administered after 16 gestational weeks is effective at reducing anemia in the later trimesters. In the wake of the obstetric transition, we observed that non-traditional causes such as pregnancy-induced hypertension has become a significant risk factor [28,35] and could be attributed to anemia of chronic disease [34], oxidative stress or placental hypoxia [28].

### Limitations and Strengths 

In interpreting these findings, we acknowledge some potential limitations. Per routine ANC practice in Ghana, Hb is measured at booking, 36 weeks and pre-delivery. Except in the second trimester where RBC indices were measured following standardized procedures, we were ethically restricted from collecting the same information at the other time points. Due to increased red cell turnover and hemodilution, which makes determining iron deficiency anemia less reliable, more sensitive methods such as serum iron, total iron-binding capacity and transferrin could have been measured, but we did not have the capacity to measure these parameters. Considering the complexity of assessing dietary intakes, we admit that diagnosing specific anemias caused by nutrient deficiencies is not as simple as presented in this paper. Most women were unaware of their pre-pregnancy body weight; hence the use of first-trimester weight to assess BMI. Dietary data were collected only once; at first ANC booking. Dietary practices could change as pregnancy advanced. Taking quantitative dietary data would have provided insight into the nutrient content of intakes. Establishing a causal relationship requires more methodologically robust designs like randomization, which is difficult in nutritional studies.

Despite these limitations, this study adds evidence on anemia at the various trimesters of pregnancy and particularly at the point of delivery which is a novel contribution. Assessing anthropometry by BMI, MUAC, weight gain and change in weight provided alternatives for monitoring nutritional status as the pregnancy progressed. Anemia is likely to be underestimated in persons residing at high altitudes and among smokers [21], but the effect was nullified in that the study was conducted in a similar geographical location and in a population where smoking was highly uncommon (<1% smoked per week). Study participants were recruited from primary, secondary and referral facilitates providing an overview of the burden in urban, peri-urban and rural populations. Unlike most studies where retrospective data are used, we collected data prospectively allowing us to explore the role of diverse factors.

## 5. Conclusions 

Fifty percent of the participants were anemic, making it a severe public health problem in Ghana with the levels no better than that reported in the 2014 demographic and health survey. Diets were energy-dense and monotonous, resulting in over one-third not meeting the FAO minimum dietary diversity indicator; a main significant risk factor for anemia in all the pregnancy trimesters. Therefore, microcytic and hypochromic anemias which point to micronutrient deficiencies were highly prevalent. Reducing anemia by 50% in women of reproductive age as per the second target of the 2012 World Health Assembly Resolution [18] will take concerted effort to achieve starting with promoting dietary diversification, food fortification, nutrition education and iron-folic acid supplementation not just for pregnant women but for all vulnerable groups.

## Figures and Tables

**Table 1:** Socio-demographic, nutritional status and health characteristics of the study participants stratified according to the adequacy of micronutrient intakes.

Variable	Sub-Groups	Overall %	Micronutrient Dense Diet	Micronutrient Poor Diet	χ^2^
Maternal age	<20 years 20–29 years 30–39 years ≥40 years	8.4 54.0 32.9 4.7	8.8 52.7 33.3 5.2	7.9 56.0 32.1 4.0	0.753
Parity	No child 1–2 children ≥3 children	29.2 51.4 19.4	27.7 50.4 21.9	31.8 53.0 15.2	0.070
Marital status	Married	73.0	76.3	68.5	0.054
Place of residence	Rural	32.8	30.8	35.7	0.335
Employment status	Employed	77.7	79.3	74.0	0.145
Educational level	None/primary Secondary Tertiary	13.8 70.9 15.3	11.6 74.0 14.5	17.0 66.7 16.4	0.212
Level of care	Primary ^a^ Secondary ^a^ Tertiary	15.4 69.4 15.2	9.1 78.6 12.3	24.4 56.4 19.2	<0.0001
Pregnancy intention	Unplanned	37.1	32.2	43.8	0.012
HIV status	Positive	2.1	2.2	1.9	0.553
Hepatitis B status	Positive	4.2	6.3	1.1	0.047
VDRL status	Positive	3.4	2.8	4.2	0.342
Malaria infection	Positive ^b^	10.3	7.2	14.5	0.081
Worm infestation	Positive	4.0	0.0	10.3	0.021
Sickling status	Positive	14.0	13.4	14.9	0.437
Blood group	A ^a^ B AB O ^a^	19.7 24.6 4.8 50.9	16.2 24.9 3.5 55.5	24.7 24.1 6.6 44.6	0.051
Rhesus status	Negative	6.6	5.7	7.8	0.263
BMI ^c^	Underweight Overweight Obese	9.6 23.9 9.4	9.7 24.1 6.2	9.5 23.7 3.2	0.786
MUAC ^c^	<24 cm	8.2	6.4	10.8	0.130
Counselled on diet	Yes	51.7	52.3	50.8	0.446
Counselled on IFA	Yes	28.8	25.7	33.1	0.105
Takes daily IFA	Yes	96.8	97.0	96.6	0.566
Food taboos	Yes	17.7	16.1	20.4	0.169

^a^ Bonferroni adjustment indicating the column proportions that differed significantly at <0.05 level. ^b^ Malaria was tested using rapid diagnostic test or blood microscopy. ^c^ BMI, body mass index was estimated only in the first trimester; whereas MUAC, mid-upper arm circumference was measured throughout pregnancy. IFA, iron-folic acid.

**Table 2:** Binary logistic regression showing the determinants for meeting the minimum dietary diversity indicator.

Variable (Reference)	Sub-Groups	Unadjusted			Adjusted		
		UOR	95% CI	*p*-Value	AOR	95% CI	*p*-Value
Age (years) (<20 years)	20–29	0.83	0.41–1.68	0.622	0.43	0.09–2.04	0.293
	30–39	0.99	0.48–2.04	0.996	0.57	0.10–3.11	0.518
	≥40	2.12	0.64–6.96	0.214	0.62	0.05–6.76	0.701
Parity (≥3 children)	1–2 children	1.61	0.92–2.83	0.098	1.93	0.50–2.36	0.821
	No child	0.83	0.54–1.25	0.382	1.95	0.71–5.33	0.192
Marital status (Cobabitating)	Married	1.61	0.95–2.72	0.076	2.46	0.94–6.40	0.065
	Single	1.5	0.73–3.07	0.269	1.96	0.51–7.47	0.322
Women’s employment (Unemployed)	Informal sector	1.93	1.16–3.21	0.011	1.49	0.54-4.14	0.436
	Formal sector	1.31	0.67–2.56	0.427	0.59	0.09–3.82	0.586
	Student	1.62	0.59–4.41	0.344	0.55	0.08–3.48	0.532
Partner’s employment (Unemployed)	Informal sector	0.86	0.28–2.65	0.803	0.24	0.03–1.55	0.136
	Formal sector	0.91	0.28–2.90	0.884	0.54	0.06–4.36	0.569
	Student	0.39	0.08–1.93	0.253	0.06	0.00–1.51	0.088
Women’s education (Up to primary)	JHS	1.23	0.70–2.17	0.465	1.07	0.40–2.84	0.877
	SHS	1.08	0.56–2.07	0.804	2.13	0.64–7.00	0.212
	Tertiary	1.02	0.51–2.03	0.944	3.74	0.49–28.60	0.203
Partner’s education (Up to primary)	JHS	1.80	0.84–3.85	0.125	1.26	0.37–4.21	0.707
	SHS	1.85	0.86–4.01	0.114	2.13	0.60–7.53	0.238
	Tertiary	1.52	0.70–3.28	0.286	0.69	0.13-3.55	0.663
Level of care (Primary)	Secondary	7.32	4.10–13.09	0.000	5.12	2.12–12.37	0.000
	Tertiary	0.72	0.30–1.68	0.448	0.80	0.20–3.09	0.747
Body mass index (Normal)	Underweight	1.65	0.80–3.37	0.171	2.76	0.77–9.83	0.097
	Overweight	0.76	0.48–1.21	0.259	0.71	0.32–1.56	0.415
	Obese	0.97	0.49–1.89	0.930	0.72	0.22–2.31	0.585
Diet counselling	Yes	1.42	0.89–2.28	0.137	1.63	0.85–3.11	0.136
IFA counselling	Yes	0.71	0.42–1.18	0.194	1.00	0.47–2.11	0.990
Pregnancy planned	Yes	1.77	1.19–2.61	0.004	2.31	1.07–4.92	0.031
Food taboos	Yes	1.31	0.80–2.14	0.267	1.09	0.49–2.44	0.821
Sickling	Positive	3.41	0.73–15.77	0.116	1.20	0.11–13.12	0.880
History of NCDs (None)	Hypertension	0.97	0.57–1.65	0.915	0.41	0.16–1.08	0.073
	Diabetes	0.58	0.21–1.54	0.278	0.32	0.04–2.53	0.285
	Both	1.44	0.49–4.24	0.506	0.95	0.19–4.57	0.953

Model summary: *n* = 364, Prob > chi2 = 0.0000; *R*^2^ = 0.2323, Log likelihood = −131.54212. UOR, unadjusted odds ratio; AOR, unadjusted odds ratio; JHS, junior secondary school (ninth grade); SHS, senior secondary school (12^th^ grade); IFA, iron-folic acid; NCDs, non-communicable diseases.

**Table 3:** Distribution of anemia by red blood cell morphology stratified according to adequacy of micronutrient intakes.

RBC Index	Reference Range^a^	Interpretation of RBC Morphology	Overall $\bar{x}$ ± SD	Overall %	Micronutrient Dense	Micronutrient Poor	*p*-Value
Hb (g/dL) ^b^	<7.0	Severe	10.80 ± 1.20	0.2	0	0.6	<0.0001
	7.0–9.9	Moderate ^c^		23.1	6.2	47.1	
	10.0–10.9	Mild		31.1	32.5	29.1	
	≥11.0	Non-anemic ^c^		45.5	61.3	23.3	
Hct (%)	<30	Low ^c^	33.72 ± 18.20	22.3	15.7	39.3	<0.0001
	30–39	Normal ^c^		74.7	80.6	59.5	
	>39	High		3.0	3.7	1.2	
RBC count (×10^12^/L)	<2.81	Low	6.75 ± 29.88	0.3	0.5	0	0.788
	2.81–4.49	Normal		81.1	80.6	82.4	
	>4.49	Polycythemia		18.6	19.0	17.6	
MCV (fl)	<85.8	Microcytic ^c^	83.45 ± 46.46	79.4	73.6	94.1	0.007
	85.8–99.4	Normal ^c^		18.9	24.1	5.9	
	>99.4	Macrocytic		1.7	2.3	0	
MCH (pg/cell)	<33	Low	27.76 ± 15.34	88.4	86.6	92.9	0.089
	30–33	Normal		11.6	13.4	7.1	
MCHC (g/dL)	<32.4	Hypochromic ^c^	33.90 ± 13.26	29.3	24.9	35.8	0.021
	32.4–35.2	Normal ^c^		47.7	54.2	38.2	
	>35.2	Hyperchromic		23.0	20.9	26.0	
RDW (%)	<12.3	Low	41.75 ± 19.71	0	0	0	0.365
	12.3–14.7	Normal		2.3	2.8	1.2	
	>14.7	High		97.7	97.2	98.8	

^a^ Morphologic types determined using references suggested by Abbassi-Ghanavati [22]. ^b^ Anemia determined using WHO classification [21]. ^c^ Adjusted Bonferroni statistics shows column proportions differ significantly at <0.05. $\bar{x}$, mean; SD, standard deviation; Hb, hemoglobin; Hct, hematocrit; RBC, red blood cell; MCV, mean corpuscular volume; MCH, mean corpuscular hemoglobin; MCHC, mean corpuscular hemoglobin concentration; RDW, red cell distribution width.

**Table 4:** Ordinal regression showing the adjusted odds for anemia in the first, second and third trimesters of pregnancy and at admission for labor and delivery.

Maternal Risk Factors (Reference in Square Brackets)		Adjusted Odds Ratio (95% Confidence Interval) for Anemia			
	Overall	1st Trimester	2nd Trimester	3rd Trimester	Pre-Delivery ^c^
Maternal age ^a^	0.90 (0.84–0.96) *	0.92 (0.86–0.98) *	0.99 (0.95–1.05)	0.94 (0.89–0.99) *	1.01 (0.94–1.09)
BMI/MUAC ^a,b^	0.87 (0.82–0.94) *	0.91 (0.85–0.98) *	0.97 (0.86–1.09)	0.99 (0.97–1.02)	0.96 (0.84–1.10)
Housewife (self/formal job)	0.31 (0.12–0.80) *	0.67 (0.27–1.69)	0.57 (0.16–2.05)	0.64 (0.24–1.72)	0.61 (0.23–1.64)
Primary education (tertiary) Secondary education (tertiary)	1.45 (0.44–4.86) 1.00 (0.38–2.63)	2.31 (0.65–8.19) 1.13 (0.40–3.18)	0.08 (0.01–0.76) * 0.03 (0.00–0.28) *	1.40 (0.36–5.47) 0.76 (0.27–2.09)	0.08 (0.01–1.25) 0.21 (0.02–2.64)
Poor diet intake (adequate diet)	2.73 (1.35–5.50) *	2.53 (1.28–4.99) *	2.99 (1.12–8.02) *	4.18 (1.97–8.87) *	1.63 (0.65–4.08) *
No dietary advice (counselled)	1.14 (0.63–2.09)	2.07 (1.07–3.98) *	1.98 (0.78–4.99)	1.48 (0.77–2.81)	3.52 (1.11–11.15) *
No IFA advice (counselled)	1.23 (0.63–2.39)	0.84 (0.42–1.67)	1.74 (0.24–12.77)	2.40 (1.13–5.09) *	1.27 (0.44–3.67)
Non-routine IFA use (daily IFA)	1.08 (0.10–11.54)	2.08 (0.42–10.17)	2.24 (0.25–19.89)	7.05 (1.06–46.81) *	3.42 (0.31–37.37)
Positive sickle cell (negative)	1.30 (0.53–3.19)	1.26 (0.53–3.03)	1.14 (0.39–3.37)	2.55 (1.07–6.08) *	1.33 (0.44–4.06)
Positive malaria (negative)	1.64 (0.52–5.17)	5.32 (1.35–20.90) *	3.42 (1.11–10.54) *	3.06 (0.84–11.18)	2.05 (0.47–8.97)
No. antenatal care visits ^a^	0.85 (0.75–0.96) *	-	0.77 (0.63–0.95) *	0.80 (0.76–0.96) *	0.76 (0.62–0.94) *
No. of IPT in pregnancy ^a^	1.06 (0.74–1.50)	-	1.02 (0.64–1.60)	0.95 (0.65–1.39)	1.01 (0.67– 1.52)
Preeclampsia (No)	0.94 (0.45–1.93)	-	0.86 (0.31–2.40)	2.71 (1.23–5.99) *	3.38 (1.17–9.76) *
Gestational diabetes (negative)	0.93 (0.41–2.10)	-	0.76 (0.32–1.79)	1.03 (0.32–3.38)	2.13 (0.54–8.35)
Model summary	*n* = 250; Prob > chi^2^ = 0.0000; *R*^2^ = 0.2205; Log likelihood = −122.113	*n* = 291; Prob > chi^2^ = 0.0000; *R*^2^ = 0.2112; Log likelihood = −161.213	*n* = 244; Prob > chi^2^ = 0.0000; *R*^2^ = 0.1963; Log likelihood = −112.009	*n* = 248; Prob > chi^2^ = 0.0000; *R*^2^ = 0.1898; Log likelihood = −129.755	*n* = 245; Prob > chi^2^ = 0.0022; *R*^2^ = 0.1858; Log likelihood = −90.505

* *p* < 0.05; ^a^ These are continuous variables; ^b^ BMI was used for the overall and first trimester models while MUAC was used in later trimesters. ^c^ This is at the point of admission for labor and delivery. BMI, body mass index; MUAC, mid-upper arm circumference; IFA, iron-folic acid; ITP, intermittent preventive treatment for malaria in pregnancy.

## Supplementary Materials

The following are available online at https://www.mdpi.com/2072-6643/12/3/777/s1, Table S1: Ordinal regression showing associations between maternal risk factors and the unadjusted odds for anemia in the first, second and third trimesters of pregnancy and at the point of admission for labor and delivery.
