# Supplementary material for: Maternal Dietary Intakes, Red Blood Cell Indices and Risk for Anemia in the First, Second and Third Trimesters of Pregnancy and at Predelivery
Source: Nutrients. 2020 Mar 15;12(3):777. doi: 10.3390/nu12030777 (PMC7146471; doi:10.3390/nu12030777)
Supplement: Supplementary file 1 [file nutrients-12-00777-s001.pdf]

Supplementary

Table S1. Ordinal regression showing associations between maternal risk factors and the unadjusted odds for anaemia in the first, second and third trimesters of pregnancy and at the point of admission for labour and delivery

Maternal risk factors (verses reference in brackets)	Unadjusted odds ratio [95% confidence interval] for maternal anaemia				
	Overall	Associations stratified by trimester in pregnancy			
		1st trimester	2nd trimester	3rd trimester	Pre-delivery
≤19 years (vs 20-34 years)	3.69 (1.83-7.41)[a]	7.42 (2.86-19.27)[a]	3.18 (1.39-7.26)[a]	2.33 (1.15-4.74)[a]	2.20 (1.07-4.53)[a]
≥35 years (vs 20-34 years)	0.79 (0.48-1.33)	0.45 (0.24-0.85)[a]	0.64 (0.34-1.20)	0.78 (0.46-1.34)	0.91 (0.52-1.62)
Parity[b]	0.86 (0.74-0.99)[a]	0.71 (0.66-0.86)[a]	0.93 (0.78-1.10)	0.89 (0.77-1.03)	0.94 (0.80-1.11)
Households size[b]	1.00 (0.92-1.09)	0.93(0.83-1.05)	1.06 (0.96-1.18)	0.98 (0.90-1.08)	0.99 (0.91-1.09)
Primary facility (vs tertiary)	3.90 (1.91-7.96)[a]	2.41 (1.04-5.56)[a]	1.36 (0.61-3.03)	4.51 (2.05-9.90)[a]	2.06 (0.91-4.64)
Secondary facility (vs tertiary)	3.71 (2.05-6.73)[a]	3.13 (1.50-6.53)[a]	1.68 (0.86-3.31)	3.77 (2.00-7.11)[a]	3.04 (1.62-5.70)[a]
Rural residence (vs urban)	1.19 (0.80-1.75)	1.29 (0.80-2.07)	1.09 (0.67-1.76)	1.04 (0.69-1.57)	1.18 (0.77-1.81)
Single (vs married)	1.76 (1.17-2.67)[a]	2.83 (1.71-4.71)[a]	1.13 (0.69-1.86)	1.56 (1.01-2.41)[a]	1.64 (1.03-2.61)[a]
Pregnancy unplanned (vs planned)	1.50 (1.03-2.19)[a]	1.93 (1.21-3.07)[a]	1.06 (0.67-1.68)	1.38 (0.93-2.05)	1.41 (0.93-2.14)
No/primary education (vs tertiary)	2.07 (1.07-4.02)[a]	3.41 (1.43-8.10)[a]	1.51 (0.67-3.38)	1.96 (0.94-4.07)	1.41 (0.67-2.97) 1.16
Secondary education (vs tertiary)	1.43 (0.85-2.40)	2.35 (1.19-4.61)[a]	0.97 (0.53-1.80)	1.53 (0.87-2.67)	(0.66-2.04)
Partner primary (vs tertiary edu.)	2.40 (1.14-5.06)[a]	2.84 (1.17-6.91)[a]	1.36 (0.57-3.26)	2.13 (0.92-4.94)	1.50 (0.59-3.80)
Partner secondary (vs tertiary edu.)	1.67 (1.10-2.55)[a]	1.65 (0.99-2.75)	1.23 (0.74-2.04)	1.76 (1.13-2.74)[a]	1.90 (1.18-3.04)[a]
Housewife (vs self/formal job)	1.34 (0.86-2.06)	2.09 (1.24-3.53)[a]	1.25 (0.74-2.11)	1.35 (0.86-2.13)	1.21 (0.75-1.95)
Underweight (vs normal BM)	1.37 (0.75- 2.52) 0.72	1.89 (0.79-4.53) 0.62	1.30 (0.61-2.79)	1.41 (0.75-2.66)	1.22 (0.64-2.33)
Overweight (vs normal BMI)	(0.46-1.12) 0.37	(0.36-1.04) 0.24 (.10-	0.81 (0.48-1.37) 0.31	0.66 (0.41-1.06) 0.45	0.76 (0.46-1.24)
Obese BMI (vs normal BMI)	(0.18-0.74)[a]	0.59)[a]	(0.12-0.79)[a]	(0.22-0.92)[a]	0.41 (0.19-0.88)[a]
Mid-upper arm circumference[b]	0.89 (0.84-0.95)[a]	0.88 (0.83-0.94)[a]	0.91 (0.86-0.97)[a]	0.92 (0.86-0.98)[a]	0.93 (0.87-0.99)[a]
Weight at pre-delivery[b]	0.98 (0.96-0.99)	-	-	0.98 (0.97-0.99)[a]	0.98 (0.97-0.99)[a]
Gestational age at registration[b]	1.03 (100-1.06)[a]	1.06 (1.02-1.10)[a]	1.03 (0.99-1.07)	1.00 (0.97-1.03)	1.01 (0.98-1.04)
No. antenatal care visits[b]	0.86 (0.78-0.94)[a]	-	0.91 (0.81-1.02)	0.87 (0.79-0.96)[a]	0.84 (0.76-0.92)[a]

Poor diet (vs adequate intake)	3.30 (2.18-4.99)[a]	3.71 (2.25-6.12)[a]	2.27 (1.40-3.66)[a]	2.89 (1.87-4.46)[a]	1.98 (1.26-3.12)[a]
No dietary advice (vs counselled)	1.14 (0.74-1.74)	1.06 (0.69-1.63)	1.09 (0.71-1.69)	1.34 (0.85-2.11)	1.19 (0.73-1.94)
No IFA advice (vs counselled)	1.14 (0.72-1.81)	1.26 (0.79-2.00)	1.04 (0.65-1.67)	1.10 (0.67-1.80)	1.34 (0.79-2.27)
Non-routine IFA use (vs daily)	0.96 (0.28-3.30)	-	1.40 (0.45-4.32)	1.78 (0.49-6.46)	1.00 (0.25-3.98)
Sickle cell trait (vs negative)	1.02 (0.52-1.99)	1.09 (0.57-2.09)	1.09 (0.57-2.08)	1.61 (0.81-3.19)	1.28 (0.62-2.62)
STI positive (vs negative) [c]	0.88 (0.41-1.89)	1.14 (0.47-2.75)	0.90 (0.35-2.27)	0.71 (0.33-1.55)	0.61 (0.26-1.45)
Malaria infection (vs negative)	3.61 (1.58-8.25)[a]	1.94 (0.67-5.65)	4.29 (1.55-11.88)[a]	3.29 (1.12-9.67)[a]	2.04 (0.83-5.01)
No. of IPTp[b]	0.89 (0.72-1.11)	-	0.85 (0.66-1.09)	0.93 (0.74-1.16)	0.94 (0.74-1.18)
Preeclampsia (vs none detected)	0.63 (0.38-1.05)	-	0.76 (0.43-1.33)	1.08 (0.63-1.85)	0.90 (0.51-1.57)
Gestational diabetes (vs negative)	1.03 (0.56-1.90)	-	1.14 (0.61-2.15)	1.03 (0.54-1.98)	0.81 (0.40-1.66)
Blood group A (vs blood group O)	1.54 (0.94-2.52)	1.05 (0.60-1.85)	1.45 (0.82-2.57)	1.21 (0.72-2.05)	2.19 (1.26-3.80)[a]
Blood group B (vs blood group O)	1.26 (0.80-1.99)	1.42 (0.82-2.46) 2.17	1.10 (0.64-1.88)	1.25 (0.78-2.00) 3.91	1.75 (1.06-2.88)[a]
Blood group AB (vs blood group O)	3.48 (1.41-8.59)[a]	(0.70-6.77)	4.23 (1.37-13.04)[a]	(1.45-10.55)[a]	2.72 (1.08-6.86)[a]

[a]p < 0.05. [b]scale variable; [c]Sexually transmitted infections included HIV, hepatitis B and syphilis. ITP, intermittent preventive treatment for malaria in pregnancy; BMI, body mass index; IFA, iron-folic acid supplementation.
